# Predictors of quality of life of medical students and a comparison with quality of life of adult health care workers in Thailand

**DOI:** 10.1186/s40064-016-2267-5

**Published:** 2016-05-10

**Authors:** Chaisiri Angkurawaranon, Wichuda Jiraporncharoen, Arty Sachdev, Anawat Wisetborisut, Withita Jangiam, Ronnaphob Uaphanthasath

**Affiliations:** Department of Family Medicine, Faculty of Medicine, Chiang Mai University, 110 Intawaroros Road, Sriphum, Muang, Chiang Mai, 50200 Thailand; Chiang Mai Medical Center Hospital, 21 Nuntaram Road, Haiya, Muang, Chiang Mai, 50100 Thailand

**Keywords:** Quality of life, Medical students, Health care workers, Thailand

## Abstract

**Introduction:**

There have been few studies which have compared the quality of life between medical students and adult health care workers.

**Aims:**

(1) To compare health related quality of life (HRQoL) between medical students and adult health care workers and (2) to identify factors associated with quality of life of medical students.

**Methods:**

A cross sectional survey of medical students at Chiang Mai University and health care workers at Chiang Mai University Hospital in 2013.

**Results:**

Compared with the population of adult health care workers, medical students had a higher physical HRQoL but similar mental HRQoL. This is potentially mediated by the presence of depression, as the prevalence of depressive symptoms was similar in both groups. Higher academic achievement and absence of underlying biomedical conditions were associated with higher HRQoL in medical students.

**Conclusion:**

The psychological burden for medical students in Thailand could be at similar levels to that of their adult health care counterparts.

## Background

Health related quality of life (HRQoL) is an important health outcome measurement since it assesses health not only on the basis of years living but in terms of quality living (Centers for Disease Control and Prevention 2014). Biological, behavioral, psychological and socioeconomic factors as well as age and gender can influence an individual’s level of health related quality of life. However, the relationships between these factors and quality of life can vary in different cultures and populations (Bowling 2005).

Medical students are considered to be a high risk population for poor quality of life (McNeill et al. 2014). Various research findings from the past few decades have shown potential negative impacts of attending medical school on student health (Paro et al. 2010; Rosal et al. 1997). Medical students are found to have higher level of overall psychological distress relative to both the general population and age matched peers (Dyrbye et al. 2006). Studying and training in a medical school causes stress from high competition, lack of free time and psychological distress from experiencing illnesses and suffering of patients (Dezee et al. 2012; Rosal et al. 1997). Depression is also a common mental health problem found among medical students but is usually not recognized (Ketumarn et al. 2013). Medical students may view mental illness as a form of weakness therefore resisting medical help (McNeill et al. 2014). All these factors may contribute to lower quality of life among medical students (Paro et al. 2010).

Quality of life among medical students has been emphasized as literature has suggested that a lower quality of life is associated with an unhealthy lifestyle, mental health problems and academic failure as well as having a negative impact on professional development (Ibrahim et al. 2014; Rosal et al. 1997).

 Previous research from western countries has shown that medical students have lower mental quality of life and more mental illness compared to the general population (Henning et al. 2012; Dyrbye et al. 2014). However, few studies have compared the quality of life between medical students and the adult health care worker population, which is another population documented as having high levels of psychological distress (Wisetborisut et al. 2014; Teles et al. 2014).

This study aims to compare physical and mental health related quality of life between medical students and health care workers and to identify sociodemographic and biomedical factors associated with quality of life among medical students. Assessment and identification of factors associated with quality of life will help guide specific interventions to promote better quality of life and to help prevent deteriorating conditions which may negatively influence the quality of life among medical students.

## Methods

A cross sectional survey was conducted among undergraduate medical students attending Chiang Mai University between March and May 2013. During orientation for the new academic year, students were asked to complete a self-reported questionnaire on demographic factors, underlying medical conditions and quality of life. Students entering the first year of medical school were excluded from the study as they had yet to start medical school. For the comparison population, during the same period, a survey among health care workers working for Chiang Mai University Hospital was also conducted (Angkurawaranon et al. 2014). This health care worker population includes all types of personnel employed by the university hospital which included doctors, dentists, nurses and lab technicians as well as a number of general workers.

### Measurement and definitions

Health related quality of life was measured using the RAND 36-item short form survey (SF-36) (Ware and Sherbourne 1992). It is used to calculate health related quality of life in eight subdomains: physical functioning (PF), bodily pain (BP), role limitations due to physical health problems (RP), role limitations due to personal or emotional problems (RE), mental health/emotional well-being (MH), social function (SF), vitality (VT) and general health perceptions (GH). These eight subdomains can be used to further calculate two main summary measures, the physical component score (PCS) and mental component score (MCS) Higher scores indicate higher health related quality of life. The SF-36 Thai version has been validated and found to be reliable among healthy populations (Lim et al. 2008).

Sociodemographic characteristics of medical students include age, sex, year of medical school, parental occupation, monthly household income, monthly personal expenses and whether they were local to Chiang Mai province. It was an assumption that local students from Chiang Mai province would have stronger social support from their families (Sreeramareddy et al. 2007). Regarding parental occupation, data was dichotomized into whether the student’s parents worked in the medical field or not. Evidence has suggested that if the parents were medical doctors the students could have a higher prevalence of psychological morbidity due to higher parental expectations (Sreeramareddy et al. 2007).

Regarding biomedical factors of medical students, both physical health and mental health were considered. For assessment of physical health, students were asked to list their known underlying history of chronic disease. The data were dichotomized into whether or not they had any known underlying chronic disease. Mental health was assessed mental using the Patient Health Questionnaire (PHQ-9). The nine questions of the PHQ-9 are based on the DSM-IV diagnostic criteria for major depression (Kroenke et al. 2001). The cut-off point of nine or more was used to define depression as this has been demonstrated as having good sensitivity and specificity in the Thai population (Lotrakul et al. 2008).

## Analysis plan

The quality of life scores among medical students and health care workers were summarized using descriptive statistics. To compare the quality of life scores between the student population and the adult health worker population, the mean quality of life scores were stratified by gender and tested using t tests. Multivariable analysis adjusted for age, sex and household income and depression was also conducted to explore the difference in QoL scores between medical students and health care workers.

Sociodemographic characteristics and biomedical factors associated with quality of life were analysed using the data collected from medical students using a t test or Chi square test. Any factors potentially associated with quality of life (p < 0.10) were considered for multivariable analyses. To identify the strongest factors associated with quality of life in medical students, all potentially significant variables were entered into a multivariable linear regression model. Each variable was then removed one by one, starting with the least significant variable until all remaining variables remained statistically significant (p < 0.05). All analysis was done using *Stata Statistical Software: Release 13* (StataCorp.)

## Results

A total of 1014 students participated in this study (68.5 % overall response rate among medical students), 53.1 % (538) of the study participants were female. Almost every medical student started attending the Faculty of Medicine immediately after they graduated from their high school. Only about one medical student per year was a graduate student with a prior bachelor’s degree. In the adult health worker population, 3204 participants completed the survey (59.7 % overall response rate among health care workers at Chiang Mai University Hospital). 77 % of health care workers who participated were female (2472). The average age of the medical students was 20.8 years (SD = 1.5) and for adult health care workers was 40.6 years (SD = 9.9) (Table 1).

**Table 1:** Demographic of participants

Medial students at Chiang Mai University		Health care workers^a^ at Chiang Mai University Hospital	
Number	1014	Number	3204
Mean age (SD)	20.8 (1.5)	Mean age (SD)	40.2 (10.7)
Year of medical school (%)		Age group (%)	
2nd year	23.8	<30	21.1
3rd year	22.0	30–40	27.4
4th year	21.9	40–50	27.3
5th year	13.7	>50	24.1
6th year	18.6		
Female (%)	53.1	Female (%)	77.1
Parental occupation^b^ (%)		Job positions^b^ (%)	
Health professionals	8.3	Health professionals	62.9
Non-health professionals	90.5	Non health professionals	37.1
Missing	1.2	Missing	0
Monthly household income in baht (%)		Monthly household income in baht (%)	
<30,000	22.3	<30,000	54.1
30,000–60,000	34.6	30,000–60,000	28.4
60,000–90,000	19.7	60,000–90,000	9.5
>90,000	21.0	>90,000	8.0
Missing	2.4	Missing	0

^a^The health care worker population includes all types of personnel employed by the university hospital which included doctors, dentists, nurses and lab technicians as well as a number of general workers

^b^Health professional include jobs such as doctors, nurses, physiotherapist and pharmacist where these occupations are involve with patient care and have professional licensing bodies; non health profession include jobs such as lawyers, administrative assistants and workers

### Comparing the quality of life between medical students and adult health care workers

Male and female medical students had significantly higher physical quality of life on all subdomains than adult health care workers (Tables 6, 7 of appendix). For male medical students, their average physical component summary score was 90.3 (SD = 13.7) while the average physical component summary score for their male health care worker counterparts was 74.5 (SD = 16.2). For female students, their average physical component summary score was 90.7 (SD = 10.6) compared to 74.8 (SD = 15.4) in adult female health care workers.

However, when mental quality of life was considered, medical students did not seem to have this large advantage. In male subjects, the mental component summary scores for students and workers were quite similar (78.6 vs 77.8, p = 0.31). In female medical students, although students had statistically higher mental component summary scores, the difference was small (80.2 vs 77.8, p < 0.01). By considering the four subdomains of mental quality of life individually, a consistent pattern emerged in both men and women. The mean quality of life scores in vitality (VT), role limitations due to emotional problems (RE) and mental health (MH) among medical students were quite similar to their adult counterparts (Table 2). This was also reflected in the prevalence of signs/symptoms of depression. The proportion presenting with such signs/symptoms were similar between male students and adult workers (12.6 vs 11.2, p = 0.52) and also between female students and female adult workers (7.4 vs 9.2 %, p = 0.01) (Table 2). In the multivariable analysis, additional adjustments for age, sex, household income and depression attenuated the differences in quality of life between medical students and adult health care workers (Table 3).

**Table 2:** Comparison in mental quality of life and prevalence of depression between medical students and health care workers by sex

Characteristic	Medical student	Healthcare worker	p value
*Men*			
Number	476	733	
Mean age	20.9 (1.5)	40.6 (9.9)	<0.01
Mental quality of life			
Mental component summary	78.6 (13.1)	77.8 (13.8)	0.31
VT/vitality	68.4 (13.9)	70.7 (13.5)	<0.01
SF/social function	90.2 (14.2)	83.4 (16.3)	<0.01
RE/role limitations due to emotional problems	86.7 (28.8)	85.7 (30.2)	0.56
MH/mental health	76.5 (13.6)	76.5 (13.4)	0.97
Depression (col %)		–	0.52
None	87.4	88.8	
Mild	10.7	8.9	
Moderate to severe	1.9	2.3	
*Women*			
Number	538	2471	
Mean age	20.8 (1.5)	40.1 (10.9)	<0.01
Mental quality of life			
Mental component summary	80.2 (11.4)	77.8 (13.2)	<0.01
VT, vitality	69.8 (11.6)	68.8 (13.3)	0.12
SF/social function	91.5 (13.6)	85.3 (15.8)	<0.01
RE/role limitations due to emotional problems	89.7 (24.3)	87.1 (27.4)	0.04
MH/mental health	78.2 (11.5)	76.2 (13.2)	<0.01
Depression (col %)			0.01
None	92.2	90.7	
Mild	6.3	8.0	
Moderate to severe	1.1	1.2

**Table 3:** Differences in mental quality of life between medical students and health care workers

Mental quality of life (QoL)	Mean differences in QoL score (students–workers)		
	Model 1	Model 2	Model 2
Mental component summary	1.68 (0.76–2.50)	1.74 (0.38–3.11)	1.22 (0.01–2.44)
VT/vitality	−0.12 (−1.06 to 0.81)	1.07 (−0.31 to 2.44)	0.65 (−0.64 to 1.93)
SF/social function	5.98 (4.90–7.08)	4.87 (3.27–6.48)	4.40 (2.90–5.90)
RE/role limitations due to emotional problems	1.52 (−0.44 to 3.48)	1.19 (−1.70 to 4.08)	0.44 (−2.30 to 3.18)
MH/mental health	1.13 (0.21–2.06)	1.02 (−0.34 to 2.39)	0.53 (−0.71 to 1.78)

Model 1: Adjusted for age and sex

Model 2: Adjusted for age, sex and household income

Model 3: Adjusted for age, sex, household income and depression

### Factors associated with quality of life among medical students

The results showed that physical quality of life among both males and females students were high. In terms of the relationships between sociodemographic factors and quality of life, females showed slightly higher mental health related quality of life than males (78.6 vs 80.2, p = 0.04). Increasing years at medical school were significantly associated with higher physical and mental health related quality of life. In the univariable analysis, parental occupation, household income, personal expense, grade point average and having families in Chiang Mai province did not show any significant associations with physical or mental health related quality of life among medical students (Table 4).

**Table 4:** Factors associated with quality of life among medical students

	Number	%	Physical QoL (SD)	p value	Mental QoL (SD)	p value
Sex				0.59		0.04
Male	476	46.9	90.3 (13.7)		78.6 (13.1)	
Female	538	53.1	90.7 (10.6)		80.2 (11.3)	
Year of medical school				<0.01		<0.01
2nd year	241	23.8	87.5 (12.1)		77.1 (12.8)	
3rd year	223	22.0	89.5 (14.0)		79.1 (12.3)	
4th year	222	21.9	92.0 (11.4)		79.5 (12.5)	
5th year	139	13.7	92.8 (11.1)		83.4 (10.9)	
6th year	189	18.6	92.1 (10.6)		79.8 (11.1)	
Parental occupation				0.57		0.99
None medical field	918	90.5	90.5 (12.2)		79.5 (12.2)	
Medical field	84	8.3	91.3 (9.5)		79.5 (11.8)	
Missing	12	1.2	–		–	
Household income				0.09		0.71
<30,000	226	22.3	90.6 (12.2)		78.7 (12.9)	
30,000–60,000	351	34.6	89.2 (13.3)		79.6 (11.1)	
60,000–90,000	200	19.7	91.2 (10.8)		79.5 (11.5)	
>90,000	213	21.0	91.7 (11.4)		80.0 (14.0)	
Missing	24	2.4	–		–	
Personal expense				0.43		0.31
<3000	209	20.6	89.5 (12.3)		78.9 (12.9)	
3000–6000	524	51.7	91.0 (11.5)		79.8 (11.4)	
6000–9000	152	15.0	90.6 (12.6)		80.1 (13.1)	
>9000	120	11.8	89.7 (14.4)		77.7 (13.2)	
Missing	9	0.9	–			
Residence				0.71		0.51
Chiang Mai	290	28.6	90.7 (11.7)		79.8 (12.2)	
Outside Chiang Mai	724	71.4	90.4 (12.3)		79.3 (12.2)	
Grade point average (GPA)				0.18		0.71
<3.0	336	33.1	89.7 (14.3)		79.6 (12.6)	
3.0–3.5	343	33.8	91.4 (11.0)		79.7 (11.9)	
>3.5	335	33.0	90.4 (10.9)		79.0 (12.1)	
Body mass index (BMI)				0.43		0.51
Underweight: <18 kg/m^2^	96	9.5	89.5 (13.4)		79.6 (12.2)	
Normal: 18–25 kg/m^2^	721	71.1	91.1 (11.2)		79.6 (12.1)	
Obese: ≥25 kg/m^2^	86	8.5	90.9 (10.7)		81.2 (10.8)	
Missing	111	10.9				
Self report underlying disease				<0.01		0.04
No	828	81.7	91.3 (11.2)		79.8 (12.0)	
Yes	186	18.3	87.0 (15.2)		77.8 (13.0)	
Depression				<0.01		<0.01
No	912	89.9	91.7 (10.6)		81.1 (10.3)	
Mild	85	8.4	81.5 (18.3)		68.2 (14.8)	
Moderate to severe	15	1.5	71.4 (19.4)		43.9 (11.6)	
Missing	2	0.2	–		–	

For associations between biomedical factors and quality of life, underlying medical and mental conditions were each significantly associated with quality of life. The results showed that students with reported underlying diseases showed slightly lower physical and mental health related quality of life than students who had not reported any underlying disease. As expected, students with signs and symptoms of depression also showed much lower physical and mental health related quality of life compared to those without (Table 4).

The multivariable analysis showed that in addition to year of medical school, self reported underlying disease, depression and GPA were each independently associated with physical health related quality of life among medical students (Table 8 of appendix). As regards mental quality of life, only year of medical school and having signs/symptoms of depression were independent factors in the multivariable analysis. Increasing year of medical school was associated with higher mental quality of life, while signs/symptoms of depression were associated with lower mental quality of life (Table 5).

**Table 5:** Factors associated with mental quality of life among medical students using multivariable linear regression

	Regression coefficient (β)	95 % confidence interval	p value
*Mental quality of life*			
Year of medical school			0.01^#^
2nd year	Reference		
3rd year	1.06	−0.91 to 3.02	0.29
4th year	1.12	−0.84 to 3.10	0.26
5th year	4.37	2.11 to 6.64	<0.01
6th year	1.56	−0.49 to 3.61	0.14
Depression			<0.01^#^
No	Reference		
Mild	−12.63	−15.01 to −10.23	<0.01
Moderate to severe	−36.31	−41.82 to −30.78	<0.01

^#^p value for trend

## Discussion

The study demonstrated that in comparison with an adult health care worker population, medical students had a higher physical HRQoL but a similar mental HRQoL. Among medical students, the higher the academic year in medical school the greater the association with higher physical and mental HRQoL. Higher GPA and absence of underlying medical conditions were associated with higher physical HRQoL while signs and symptoms of depression were associated with lower physical and mental HRQoL.

The identification of the trend that medical students have a higher physical QoL than adult health care workers was expected due to the higher risk of morbidities associated with increasing age (Rusli et al. 2008). However, similar levels in mental quality of life between medical students and adult health care personnel could be a potential issue that needs further research. In most subdomains of mental QoL, the QoL scores were similar between groups. This is perhaps mediated by the presence of depression, as the prevalence of depressive symptoms was similar in both groups. The results of this study also support this hypothesis as additional adjustment for depression diluted the differences in quality of life between students and workers. It is usually expected that given a similar setting and environment, mental HRQol of young adults would usually be better than their older adult counterparts (Rustoen et al. 2005; Jorngarden et al. 2006). These results suggested that the level of psychological burden on medical students could be similar to that of their adult health care counterpart.

As for factors associated with quality of life among medical students, many studies have reported that HRQoL of medical students can vary by class year (Sreeramareddy et al. 2007; Rosal et al. 1997). However, trends in associations seem to differ between settings. Literature suggested that attending medical school has a major negative impact during later clinical years as the students have to make contact with patients and this may lead to intense emotional experiences, increasing levels of stress and depression (Paro et al. 2010; Dyrbye et al. 2006). Other studies, however, have suggested that there was no significant difference from year to year (Sreeramareddy et al. 2007; Tempski et al. 2012). This study found that a higher academic year in medical school was associated with higher mental and physical HRQoL. This is consistent with the findings from a study conducted in Pakistan, which suggested that exposure to more real life situations in later clinical years may help students develop and improve coping strategies while the fear of examinations becomes somewhat subdued because of their past experience with them (Naseem and Iqbal 2010). The study also found that a higher GPA was associated with higher QoL. Reverse causation may play a role in this observed association as studies have suggested that students with a higher HRQol usually perform better academically (Hettiarachchi et al. 2014).

The study found that physical and psychological morbidities, particularly depression, were associated with lower QoL in medical students. This is supported by previous literature where chronic health problems and depressive symptoms were associated with lower HRQoL (Paro et al. 2010; Michelson et al. 2000). For decades it has been documented that depression is a common mental health problem among medical students which can affect the student’s performance as well as personal and professional development (Paro et al. 2010; Rosal et al. 1997; McNeill et al. 2014).

The study had several limitations. The study was cross sectional, thus causal relationships could not be demonstrated between the associations seen. Underlying causes and issues on why medical students may have high levels of psychological burden need to be further explored. The study achieved a high response rate to the surveys but the findings may not be entirely generalizable to other medical students in different settings due to sociocultural variations, different ethnic populations, different curriculums and entry route of medical students. Most of the medical students in Thailand attend medical school directly after completion of high school and all are Thais.

Despite its limitations, the study provides some insight into physical and mental HRQoL of medical students and their associated factors. The psychological burden of medical students in Thailand could be similar to that of their adult health care counterpart, which is already considered a population exposed to high levels of stress (Ruotsalainen et al. 2014). Despite the diversity between settings, the overall HRQoL and the proportion of medical student with depression in the study are comparable with other previous reports (Paro et al. 2010; Dyrbye et al. 2006) These findings suggest that specific preventative measures should be considered by both the Thai educational council and medical schools to improve mental HRQoL among medical students. The measures could potentially include changes in the curriculum towards more problem-based and self directed learning (Wilson et al. 1996). Interventions to improve overall mental health and develop student resilience such as group discussion, enhancing relationships between students and teachers, lectures on stress and time management may be helpful. Counseling services have been found to be effective in reducing psychological stress in some similar cases (Sharif and Armitage 2004; Velayudhan et al. 2010).

An example of a comprehensive program to improve medical students’ resilience and health in Australia is describe by Hassed et al. (2008). The interventions were based on the ESSENCE lifestyle model (Hassed 2005) which focuses on important factors such as stress management, mindfulness practice, proper exercise and healthy diets. The interventions were delivered through lectures and group discussions. Core skills and contents were assessed while students were also encouraged to maintain personal practices by keeping journals. The concepts described in the ESSENCE model have been applied in Chiang Mai University Medical School since 2010. During orientation of the new school year, students undergo the Medical Student Healthy Day activities which include taking a physical examination and a physical fitness test. Anonymized data is also collected on diet, physical activity level, smoking, alcohol use and sexual behavior. This is then used to plan population level interventions to reduce risky behaviors among medical students. However, maintaining healthy behavior and practices among medical students remains a challenging issue and one initiative, the Medical Student Healthy Day activities, has not yet been formally assessed. Nonetheless, it has been noted that the distress among physicians usually has its origins in medical school (Dyrbye et al. 2006). Therefore, such measures and interventions in this period may help prevent and reduce suffering from mental illness in medical students, improve their mental HRQoL and enable them to have better performance as well as enhance personal and professional development.

## Appendix

See Tables 6, 7 and 8.

**Table 6:** Comparison in physical quality of life between medical students and health care workers by sex

Characteristic	Medical student (n = 476)	Healthcare worker (n = 733)	p value
*Men*			
Physical quality of life			
Physical component summery	90.3 (13.7)	74.5 (16.2)	<0.01
BP/bodily pain	84.1 (18.0)	74.2 (18.2)	<0.01
PF/physical function	90.5 (15.1)	75.8 (20.9)	<0.01
RP/role limitations due to physical problems	91.7 (21.8)	84.4 (29.7)	<0.01
GH/general health	73.5 (17.3)	65.8 (16.5)	<0.01

Characteristic	Medical student (n = 538)	Healthcare worker (n = 2471)	p value
*Women*			
Physical quality of life			
Physical compoenet summery	90.7 (10.6)	74.8 (15.4)	<0.01
BP/bodily pain	83.3 (16.8)	72.8 (19.2)	<0.01
PF/physical function	90.2 (12.9)	76.6 (18.9)	<0.01
RP/role limitations due to physical problems	94.1 (18.2)	86.4 (27.9)	<0.01
GH/general health	73.2 (15.8)	64.5 (16.7)	<0.01

**Table 7:** Differences in physical quality of life between medical students and health care workers

Physical quality of life (QoL)	Mean differences in QoL score (students–workers)		
	Model 1	Model 2	Model 2
Physical component summery	15.8 (14.7–16.8)	8.42 (6.91–9.94)	8.00 (6.56–9.43)
BP/bodily pain	10.6 (9.01–11.9)	7.15 (5.21–9.08)	6.74 (4.86–8.63)
PF/physical function	13.9 (12.6–15.2)	2.32 (0.58–4.16)	1.98 (0.17–3.78)
RP/role limitations due to physical problems	6.99 (5.12–8.87)	0.62 (−2.13 to 3.38)	−0.03 (−2.70 to 2.62)
GH/general health	8.51 (7.34–9.69)	5.99 (4.25–7.72)	5.56 (3.91–7.22)

Model 1: Adjusted for age and sex

Model 2: Adjusted for age, sex and household income

Model 2: Adjusted for age, sex, household income and depression

**Table 8:** Factors associated with physical quality of life among medical students using multivariable linear regression

	Regression coefficient (β)	95 % confidence interval	p value
*Physical quality of life*			
Year of medical school			<0.01^#^
2nd year	Reference		
3rd year	2.80	0.58–5.01	0.01
4th year	4.92	2.69–7.15	<0.01
5th year	5.36	2.80–7.92	<0.01
6th year	5.20	2.83–7.57	<0.01
GPA			0.02^#^
<3.0	Reference		
3.0–3.5	1.87	0.10–0.64	0.04
>3.5	2.65	0.70–4.59	0.01
Self report underlying disease			
No	Reference		
Yes	−3.68	−5.51–1.85	<0.01
Depression			<0.01^#^
No	Reference		
Mild	−9.39	−11.94–6.83	<0.01
Moderate to severe	−18.14	−24.00–12.28	<0.01

^#^p value for trend
